# Rising Cardiometabolic Comorbidity and Inpatient Resource Utilization Among Hospitalized Patients with Hepatocellular Carcinoma, 2018–2022

**DOI:** 10.3390/jcm15062386

**Published:** 2026-03-20

**Authors:** Muhammad Haris Latif, Ayesha Kang, Eman Mazhar, Kahee Amedi, Joel Riley, Hani-El Halawany, Kamran Qureshi

**Affiliations:** 1 Department of Internal Medicine, SSM Health St. Mary’s Hospital, St. Louis, MO 63117, USA; 2 Department of Internal Medicine, Nishtar Medical University, Multan 66000, Pakistan; 3 Department of Gastroenterology, SSM Health DePaul Hospital, Bridgeton, MO 63044, USA; 4 Department of Gastroenterology, Saint Louis University Hospital, St. Louis, MO 63104, USA

**Keywords:** hepatocellular carcinoma, inpatient outcomes, national inpatient sample, metabolic dysfunction-associated steatotic liver disease, healthcare utilization

## Abstract

**Background**: Hospitalizations for hepatocellular carcinoma (HCC) increasingly reflect a complex interplay among chronic liver disease, cardiometabolic comorbidities, and systemic complications, which now exert greater influence on patient outcomes than tumor-specific factors alone. Despite this shift, contemporary data regarding the impact of the evolving comorbidity burden on inpatient resource utilization and procedural care remain limited. This study examines national trends in inpatient characteristics, procedural utilization, and outcomes among patients hospitalized with HCC between 2018 and 2022. **Methods**: A retrospective, cross-sectional analysis of adult hospitalizations was performed using the National Inpatient Sample (NIS) from 2018 to 2022. Hospitalizations involving HCC were identified through ICD-10 diagnosis codes, encompassing both principal and secondary diagnoses. Survey-weighted analyses were used to estimate national prevalence, in-hospital mortality, length of stay (LOS), and total hospital charges. Temporal trends were evaluated using survey-weighted logistic or linear regression, with calendar year as a continuous variable. Multivariable survey-weighted logistic regression models were constructed to identify adjusted predictors of inpatient mortality and procedural utilization, including liver transplantation, hepatic resection, and transjugular intrahepatic portosystemic shunt (TIPS) placement. **Results**: During the study period, an estimated 275,000 HCC-related hospitalizations occurred nationwide. The prevalence of cardiometabolic comorbidities increased significantly over time (all *p* < 0.001), including MASLD (6.6% to 8.7%), obesity (10.6% to 13.7%), diabetes (36.0% to 38.9%), and dyslipidemia (26.4% to 34.4%). In-hospital mortality rose from 8.82% (95% CI, 8.40–9.24%) in 2018 to 9.23% (95% CI, 8.81–9.65%) in 2022, with the highest rate in 2020 (9.42%). In parallel, inpatient resource utilization rose, as reflected by longer lengths of stay and higher hospitalization charges. Utilization of diagnostic endoscopic procedures, such as esophagogastroduodenoscopy and endoscopic retrograde cholangiopancreatography, increased, whereas rates of definitive inpatient oncologic and portal hypertension-directed interventions—including liver transplantation, hepatic resection, and TIPS—remained low and stable. In-hospital mortality was independently associated with markers of hepatic decompensation and systemic illness, including hepatic encephalopathy, acute kidney injury, sepsis, and hepatorenal syndrome. These associations were stronger than those observed for tumor-directed procedures, as reflected by inpatient procedural utilization patterns. **Conclusions**: Between 2018 and 2022, inpatient resource utilization among patients hospitalized with hepatocellular carcinoma increased in parallel with rising cardiometabolic comorbidity. It was primarily driven by management of hepatic decompensation and systemic illness rather than oncologic intervention. These findings characterize the evolving complexity of HCC hospitalizations in the contemporary inpatient setting.

## 1. Introduction

Hepatocellular carcinoma (HCC) is the most prevalent primary liver malignancy and remains a major contributor to cancer-related mortality worldwide. Global Cancer Statistics 2020 ranks HCC as the sixth most frequently diagnosed cancer and the third leading cause of cancer-related death worldwide. This underscores its aggressive clinical course and significant public health implications [1]. In the United States, the burden of HCC on healthcare systems is rising. This is due to increasing chronic liver disease, greater comorbidity complexity, and higher rates of hospitalization-related morbidity and mortality [1].

The epidemiology of HCC has shifted significantly. Direct-acting antiviral therapy has lowered HCV-related HCC incidence at the population level. However, hospitalized cohorts may still reflect delayed effects from prior disease burden [2,3]. At the same time, metabolic dysfunction-associated steatotic liver disease (MASLD) and alcohol-associated liver disease have become leading HCC causes [4,5]. This change parallels broader increases in obesity, diabetes, and cardiometabolic disease. These trends carry implications for inpatient outcomes and resource use [6].

Most HCC cases develop in the setting of cirrhosis. Inpatient outcomes are tied more to the severity of hepatic dysfunction than tumor-specific factors alone [7]. Hospitalizations for HCC often feature complications like hepatic encephalopathy, ascites, hepatorenal syndrome, acute kidney injury, gastrointestinal bleeding, and sepsis [8,9]. These issues result in longer hospital stays, greater healthcare costs, and higher mortality [10]. Patterns of hospitalization, therefore, offer valuable insights into managing advanced liver disease beyond tumor progression.

Earlier analyses of the National Inpatient Sample (NIS) tracked trends in HCC hospitalizations, mortality, length of stay, and costs—mostly during the pre-direct-acting antiviral (DAA) era [11,12]. These studies reported an increasing inpatient burden but did not address the recent shift to metabolic liver disease or the impact of COVID-19-related healthcare disruptions. Contemporary data are still lacking on how changes in comorbidity, organ dysfunction, and hospital-level factors affect in-hospital mortality in HCC patients.

This study uses National Inpatient Sample data from 2018 to 2022. It provides a current, nationally representative analysis of HCC hospitalizations in the United States. This period is defined by declining viral hepatitis, rising metabolic liver disease, and disruptions from the COVID-19 pandemic. The objectives are to (1) evaluate national trends in HCC-related hospitalizations and patient characteristics in the post-DAA era, (2) assess inpatient demographics, disease burden, procedural use, and resource consumption, and (3) identify predictors of in-hospital mortality among hospitalized HCC patients amid contemporary metabolic disease.

## 2. Materials and Methods

### 2.1. Study Design and Data Source

A retrospective, cross-sectional analysis was conducted using the National Inpatient Sample (NIS) from 2018 to 2022. The NIS, developed by the Healthcare Cost and Utilization Project (HCUP) and sponsored by AHRQ, is the largest publicly available all-payer inpatient database in the United States, representing a 20% stratified sample of community hospital discharges nationwide [13]. Each dataset includes demographics, hospital characteristics, diagnoses, procedures, outcomes, and resource utilization. Survey weights, strata, and cluster variables were applied according to HCUP guidance [14]. The study utilized de-identified, publicly available data from the Healthcare Cost and Utilization Project National Inpatient Sample (NIS). It was exempt from institutional review board approval under applicable U.S. regulations.

### 2.2. Study Population

All adult (≥18 years) hospitalizations in the NIS from 2018 to 2022 were evaluated. The study population included admissions with a diagnosis of hepatocellular carcinoma (HCC). These were identified using a manually defined binary variable derived from the ICD-10 diagnosis fields. Hospitalizations were identified using ICD-10-CM code C22.0 [2].

Both principal and secondary diagnoses were included to capture all encounters with HCC. This was done whether HCC was the primary reason for hospitalization or a comorbid condition. This approach enabled the identification of a broad spectrum of encounters, including complications of malignancy beyond cancer-directed care. However, this method may introduce heterogeneity. The severity and clinical context of secondary diagnoses can vary.

Hospitalizations were excluded if age, sex, discharge weight, or hospital stratum were missing. Additionally, pediatric admissions (age < 18 years) and obstetric admissions were excluded to maintain an adult-only cohort. After application of inclusion and exclusion criteria, the analytic cohort represented a nationally weighted estimate of adult HCC hospitalizations across U.S. hospitals from 2018 to 2022.

### 2.3. Demographics and Hospital Characteristics

Demographic variables included age, sex, and race/ethnicity (White, African American, Hispanic, Asian/Pacific Islander, Native American, and Other). Other variables included primary payer (Medicare, Medicaid, private insurance, self-pay, or other) and median household income quartile by ZIP code. Hospital-level variables were location (rural, urban non-teaching, urban teaching), bed size (small, medium, large), and U.S. Census region (Northeast, Midwest, South, West).

### 2.4. Clinical Covariates

Comorbidities were identified using HCUP comorbidity and diagnosis flags. ICD-10-based CCSR codes were also used. The following conditions were extracted: cirrhosis, viral hepatitis (HBV or HCV), MASLD, diabetes mellitus, hypertension, CKD, CHF, CAD, dyslipidemia, obesity, and alcohol use disorder.

For all hospitalizations with HCC (ICD-10-CM C22.0 in any diagnosis field), the principal diagnosis (I10_DX1) identified the primary cause of admission. Causes were grouped using the Clinical Classifications Software Refined (CCSR) system, version 2021.1. Groupings formed clinically meaningful categories: primary HCC, liver-related complications (such as hepatic failure, ascites, variceal bleeding, encephalopathy), infections or sepsis, cardiovascular conditions, and other systemic causes. This approach enabled evaluation of the most common admission etiologies among patients with known HCC.

Hospitalizations were classified as metabolic dysfunction-associated steatotic liver disease (MASLD) if ICD-10-CM codes for hepatic steatosis or steatohepatitis were present. At least one metabolic risk factor was required. Concurrent viral hepatitis, alcohol-related, or autoimmune liver disease had to be absent. The NIS does not provide biochemical or imaging confirmation.

### 2.5. Outcomes

The primary outcome was in-hospital mortality. Secondary outcomes included length of stay (LOS), total hospital charges (TOTCHG), and use of inpatient procedures. These procedures included endoscopy, hepatic resection, liver transplantation, and TIPS placement.

### 2.6. Procedural Identification

Endoscopic and hepatobiliary procedures were identified using CCSR procedure groupings. These included diagnostic and interventional esophagogastroduodenoscopy (EGD), colonoscopy, ERCP, TIPS, hepatic resection, and liver transplantation. Diagnostic and therapeutic endoscopic procedures were evaluated separately to characterize procedural intensity. Liver transplantation was identified using ICD-10-PCS procedure codes during the index hospitalization. ICD-10-CM diagnosis codes indicating transplant status or aftercare were also used. The NIS does not distinguish transplant eligibility or oncologic intent. In this study, liver transplantation reflects inpatient transplant-related utilization, not definitive curative therapy for HCC.

### 2.7. Statistical Analysis

All analyses accounted for the NIS complex survey design using the provided discharge weights (DISCWT), hospital strata (NIS_STRATUM), and cluster variables (HOSP_NIS) to produce national estimates. Descriptive statistics summarized demographic and clinical characteristics across study years.

- Categorical variables were compared using the Rao–Scott adjusted χ^2^ test and presented as weighted percentages.
- Continuous variables (e.g., LOS and hospital charges) were compared using survey-weighted linear regression and reported as means with standard errors.
- Temporal trends were evaluated using survey-weighted logistic or linear regression with calendar year as a continuous predictor.

To identify independent predictors of in-hospital mortality, multivariable survey-weighted logistic regression models were used. Covariates included demographics, payer type, income quartile, hospital region, and significant comorbidities (cirrhosis, viral hepatitis, MASLD, diabetes, CKD, CAD, CHF, obesity). Common inpatient complications (liver failure, hepatic encephalopathy, sepsis, renal failure, gastrointestinal bleeding, and cardiovascular events) were also included. Model selection and covariates were guided by clinical relevance and existing literature [15,16]. Multicollinearity was assessed using variance inflation factors, all of which were less than 2.0. Odds ratios (aORs) with 95% confidence intervals (CIs) were reported. Two-tailed *p*-values <0.05 were considered statistically significant.

Analyses were performed using Stata/MP version 17.0.

## 3. Results

### 3.1. Inpatient Prevalence of Hepatocellular Carcinoma

After applying inclusion and exclusion criteria, the analytic sample represented approximately 33.1 million adult hospitalizations nationwide during the study period. Among these, HCC accounted for an estimated 0.25–0.28% of all discharges, corresponding to ~55,000–58,000 hospitalizations annually.

### 3.2. In-Hospital Mortality

In-hospital mortality showed a significant upward trend from 8.82% (95% CI, 8.40–9.24%) in 2018 to 9.23% (95% CI, 8.81–9.65%) in 2022 (*p* < 0.001). The highest rate occurred in 2020 (9.42%), coinciding with the COVID-19 pandemic, followed by a modest decline in 2021 (9.14%) and a subsequent rise in 2022. Survey-weighted annual mortality trends are summarized in Table 1.

### 3.3. Comorbidities Among Patients Hospitalized with HCC

**Figure 1:**
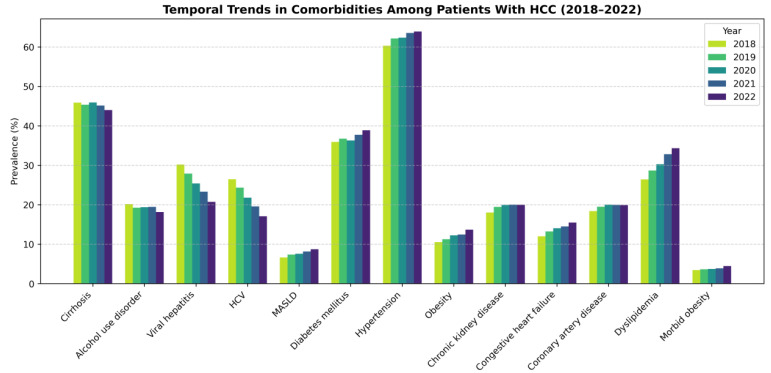
Survey-weighted temporal trends in comorbidities among hospitalized patients with hepatocellular carcinoma, 2018–2022. The graph illustrates the diverging trends between declining viral hepatitis (HCV, HBV) and rising metabolic comorbidities (MASLD, diabetes, obesity, dyslipidemia, chronic kidney disease, congestive heart failure). Cirrhosis and alcohol use disorders remained relatively stable. All temporal trends were statistically significant (*p* < 0.001). HCV: hepatitis C virus; MASLD: metabolic dysfunction-associated steatotic liver disease.

Over the study period, cardiometabolic comorbidities—including MASLD, obesity, diabetes, dyslipidemia, chronic kidney disease, and congestive heart failure—increased, while viral hepatitis and alcohol use disorder declined (Table 2 and Figure 1). Cirrhosis prevalence remained relatively stable. COVID-19 was identified in 2–5% of all HCC hospitalizations, with the highest mortality observed during the early pandemic years. In 2020 and 2021, both primary and secondary COVID-19 diagnoses were strongly associated with increased inpatient mortality.

### 3.4. Burden of Primary Hospital Admissions

**Figure 2:**
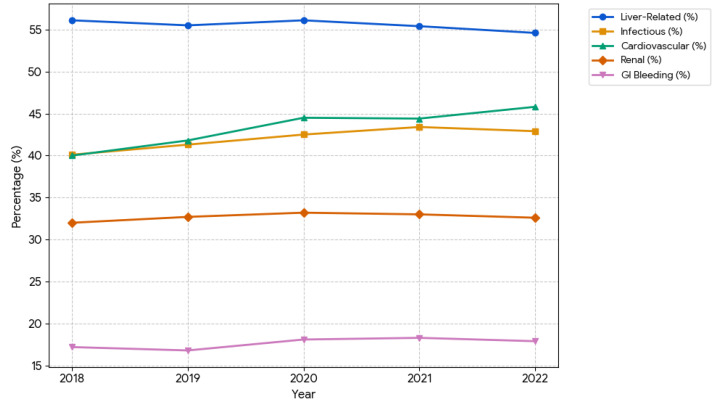
Survey-weighted temporal trends in major primary admission categories among patients hospitalized with hepatocellular carcinoma in the United States, 2018–2022. GI: gastrointestinal.

Liver-related complications remained the most common cause of hospitalization, followed by infectious and cardiovascular causes, with modest increases in admissions for these causes over time (Table 3 and Figure 2). Because admission categories were not mutually exclusive, percentages reflect co-occurring drivers of hospitalization rather than mutually exclusive primary diagnoses. Most temporal changes in comorbidity prevalence were statistically significant (*p* < 0.001).

### 3.5. Predictors of In-Hospital Mortality

Between 2018 and 2022, multivariable logistic regression identified advanced age, liver-related complications, and sepsis as the strongest independent predictors of in-hospital mortality among patients with hepatocellular carcinoma (Table 4).

### 3.6. Length of Stay and Total Hospital Charges

Between 2018 and 2022, mean hospital length of stay and total hospital charges varied significantly by primary admission category (Table 5). Infectious, liver-related, and gastrointestinal bleeding admissions had the most extended hospitalizations, while cardiovascular, renal, and other causes showed shorter and relatively stable stays over time.

Total hospital charges increased across all primary admission categories over the study period, with the highest charges consistently observed for admissions related to infectious diseases and gastrointestinal bleeding. Liver-related hospitalizations also demonstrated a steady rise in charges over time, paralleling increasing length of stay. Cardiovascular and renal admissions were associated with comparatively lower but progressively increasing charges (Table 5).

### 3.7. Demographics

Overall, hospitalized patients with hepatocellular carcinoma were predominantly older, male, and Medicare-insured, with stable racial, regional, and hospital-level distributions over time. Detailed demographic and socioeconomic characteristics across study years are presented in Table 6.

### 3.8. Temporal Trends in Endoscopic Procedures

Diagnostic endoscopic utilization, including EGD and ERCP, increased modestly over time, whereas interventional endoscopy, hepatic resection, liver transplantation, and TIPS utilization remained low and stable (Table 7).

### 3.9. Adjusted Predictors of Liver Transplantation, Hepatic Resection, and TIPS Placement

Across all three procedures, distinct patterns emerged in the adjusted predictors of inpatient liver transplantation, hepatic resection, and TIPS placement. Liver transplantation was associated with younger age, private insurance, urban teaching hospitals, and liver disease etiologies such as MASLD, hepatitis C, and alcohol-associated liver disease. Cirrhosis increased the likelihood of transplantation but was also the strongest predictor for TIPS placement, which remained a rare, highly selective event throughout the study years. Hepatic resection was more frequent among patients with MASLD, obesity, dyslipidemia, and those at urban teaching hospitals. Still, it was less likely in individuals with cirrhosis, chronic kidney disease, or alcohol associated liver disease. Notably, substance use disorder, Medicaid, and self-pay status lowered the odds of transplantation. These patterns underscore how patient characteristics and hospital factors shape the likelihood of each procedure in hospitalized HCC patients, as detailed in Table 8.

## 4. Discussion

In this nationally representative analysis of U.S. hospitalizations from 2018–2022, we found that hepatocellular carcinoma (HCC) accounted for approximately 0.25–0.28% of all adult inpatient discharges, translating to roughly 55,000–58,000 HCC-associated hospitalizations annually. Over these five years, we observed a statistically significant rise in inpatient HCC prevalence and a concurrent increase in in-hospital mortality, with the highest mortality in 2020. Although year-to-year mortality differences were statistically significant, absolute changes were modest, suggesting relative stability in inpatient HCC mortality during the study period. We further demonstrate a shifting inpatient comorbidity landscape characterized by declining viral hepatitis and increasing metabolic comorbidity, alongside a rising burden of infectious and cardiovascular admission drivers. Finally, we identify that in-hospital mortality is strongly determined by acute severity features, including hepatic encephalopathy, liver failure, hepatorenal syndrome, acute kidney injury, and sepsis, highlighting the central role of multi-organ complications in driving short-term outcomes among hospitalized HCC patients. However, the retrospective and cross-sectional nature of this analysis limits the ability to establish temporal or causal relationships, and residual confounding may persist despite the use of survey weights and multivariable adjustment.

Earlier NIS studies documented rising HCC hospitalization volume and charges. Jinjuvadia et al. (2002–2011) emphasized the growing inpatient burden [15], Mishra et al. (2005–2009) reported increasing charges and hospital outcomes [16], and Wakil et al. (2011–2017) provided pre-COVID mortality, LOS, and charge baselines [11]. Our study extends this epidemiology into 2018–2022, capturing the post-DAA and COVID-19 periods.

The primary contribution of our study is that it extends national inpatient HCC epidemiology into the contemporary period (2018–2022), spanning the post-direct-acting antiviral (DAA) era and the COVID-19 healthcare disruption period. Prior inpatient trend papers essentially end before 2018 and therefore do not capture (1) the ongoing etiologic transition toward metabolic liver disease-associated HCC, and (2) the acute pandemic-era inflection that appears in our mortality curve.

Importantly, in-hospital mortality in the NIS reflects hospitalized-case fatality rather than population-level survival. The observed mortality peak in 2020–2021 among HCC admissions was primarily attributable to concurrent COVID-19 infection. Approximately 2–5% of HCC hospitalizations involved COVID-19, and both primary and secondary diagnoses were associated with a two- to threefold increase in in-hospital mortality during the early pandemic years. By 2022, COVID-related mortality had attenuated, consistent with reduced viral virulence and improved clinical adaptation. Several mechanisms likely contributed to the upward trend and the pandemic-era mortality peak. First, multiple studies have documented delays and disruptions in HCC surveillance, diagnosis, and treatment during early COVID-19 waves, which may have shifted presentations toward more advanced disease and higher acuity among hospitalized patients [17]. Second, hospital resource constraints and modified care pathways during 2020 likely influenced admission thresholds, potentially selecting more severely ill individuals. Although our dataset cannot directly assess tumor stage or outpatient treatment deferrals, the temporal association with the early pandemic period aligns with prior reports of delayed oncologic care and disrupted HCC management [8].

A key finding is the substantial reduction in viral hepatitis and hepatitis C prevalence among hospitalized HCC patients over time, alongside increasing MASLD prevalence and increasing cardiometabolic comorbidity (e.g., obesity, dyslipidemia, diabetes). This mirrors the broader epidemiologic literature, which describes declining HCV-related HCC risk following DAA implementation and a rising share of NAFLD/MASLD-related HCC in the United States [18]. The “changing epidemiology” framework for HCC has been well described, with metabolic liver disease increasingly driving incident HCC as viral hepatitis becomes more treatable at scale.

Notably, metabolic dysfunction-associated steatotic liver disease (MASLD) was associated with lower odds of in-hospital mortality in multivariable analyses. This association should be interpreted cautiously as administrative data that cannot fully account for disease severity, admission indication, or competing risks. This finding does not imply an intrinsic protective effect of MASLD. Instead, it likely reflects differences in baseline hepatic reserve, portal hypertension severity, and admission acuity compared with viral- or alcohol-related cirrhosis phenotypes. In hospitalized patients with hepatocellular carcinoma, short-term outcomes are often driven more by decompensated cirrhosis and systemic complications than by tumor biology alone, and observed comorbidity associations may therefore reflect residual confounding by disease severity not fully captured in administrative datasets [5].

Although MASLD is now recognized as one of the fastest-growing etiologic drivers of hepatocellular carcinoma, prior observational studies have similarly reported a paradoxical association between MASLD-related HCC and lower short-term or in-hospital mortality compared with other etiologies. One potential explanation is that patients with MASLD-associated HCC are more frequently hospitalized for nonhepatic indications, such as cardiovascular or infectious events, rather than acute hepatic decompensation, which may contribute to lower observed inpatient fatality rates [10]. In addition, this pattern parallels the well-described “obesity paradox,” wherein overweight and obese patients demonstrate improved short-term survival in hospitalized and chronic disease cohorts despite increased long-term risk, a phenomenon reported across cardiovascular disease, cirrhosis, and other medically complex populations [11].

Liver-related complications remained the most common admission category (≈55%). Still, we observed a notable increase in cardiovascular and infectious admission drivers over time, while renal and GI bleeding burdens remained relatively stable. These patterns reinforce that the inpatient HCC population increasingly represents a medically complex cohort with competing comorbidities and multi-system complications. The rising cardiovascular burden aligns with the increasing prevalence of metabolic comorbidity observed in our cohort and reflects the well-recognized overlap between MASLD and cardiometabolic disease. Admission categories were not mutually exclusive; individual hospitalizations could contribute to more than one etiology category if multiple risk factors were coded. Outcomes in 2020–2021 may reflect transient disruptions in care during the COVID-19 pandemic, which were not explicitly modeled as interaction terms.

The infectious burden—particularly sepsis-related admissions—may also reflect cirrhosis-associated immune dysfunction and high vulnerability to systemic infection in advanced liver disease [19]. The modest rise in infectious admissions during and after 2020 plausibly reflects both baseline vulnerability and pandemic-related shifts in healthcare utilization and infection epidemiology [8].

Over the years, hepatic encephalopathy, liver failure, hepatorenal syndrome, acute kidney injury, and sepsis were the strongest predictors of in-hospital mortality in our models. This is consistent with the clinical reality that hospitalized HCC patients frequently die from complications of end-stage liver disease or systemic illness rather than from oncologic progression alone. Prior inpatient analyses have similarly linked mortality risk to severity markers and hospital factors [5].

**Figure 3:**
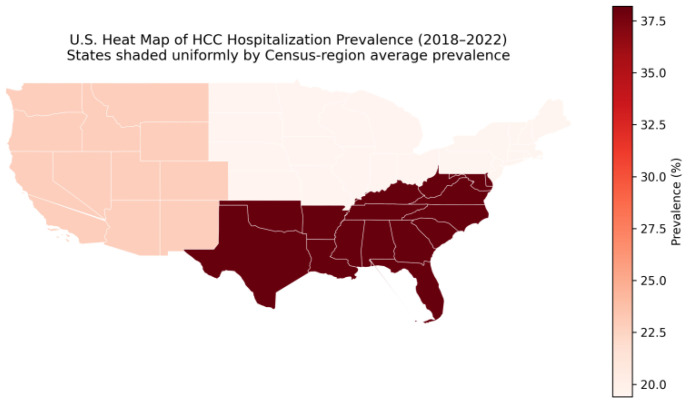
U.S. Heat Map of Census Region–Averaged Hospitalization Prevalence for Hepatocellular Carcinoma, 2018–2022.

We also observed associations between mortality and payer status and race/ethnicity, suggesting persistent disparities in inpatient outcomes (Figure 3). Although this analysis is descriptive, the disproportionate concentration of hospitalizations in the Southern United States is consistent with prior reports of higher chronic liver disease prevalence, metabolic risk burden, and hepatocellular carcinoma incidence in this region [20]. Although hospitalizations were disproportionately concentrated in the Southern United States, lower adjusted in-hospital mortality in this region may reflect case-mix differences, referral patterns to high-volume centers, or residual confounding related to admission thresholds and disease severity. While administrative datasets cannot fully disentangle mechanisms, these findings are directionally consistent with broader disparities in the literature in HCC care (screening, stage at diagnosis, and access to specialized treatment), which continues to motivate health-systems interventions aimed at equitable prevention and early detection.

We found increasing total hospital charges across admission categories, with infectious and GI bleeding admissions consistently among the highest-cost and longest-stay hospitalizations. Prior NIS-based studies have similarly described the substantial financial burden of inpatient HCC care and its upward trajectory over time [5]. Our study adds contemporary estimates through 2022, which are essential given ongoing inflation, increasing procedural intensity, and rising comorbidity complexity—factors that are likely to amplify inpatient costs in the modern era.

Increased inpatient procedural burden was driven by diagnostic endoscopy and decompensation management rather than oncologic interventions. While diagnostic EGD and ERCP increased modestly, liver transplantation, hepatic resection, and TIPS remained low and stable. This aligns with prior studies showing that HCC hospitalizations are precipitated by cirrhosis complications—bleeding, infection, renal dysfunction—rather than tumor-directed therapy [7]. Increased diagnostic endoscopy likely reflects heightened vigilance in an increasingly comorbid population. Procedural utilization findings are descriptive and intended to contextualize real-world inpatient practice patterns rather than infer causality. Liver transplantation remained concentrated at urban teaching hospitals and among privately insured patients [21,22], with persistent socioeconomic and institutional disparities despite stable overall rates.

The contrasting predictors of hepatic resection versus transplantation underscore biologically plausible selection patterns. Resection was favored in patients with metabolic-associated disease and preserved hepatic function, whereas transplantation was driven by advanced cirrhosis and decompensation. The strong inverse association between cirrhosis and resection is consistent with surgical guidelines emphasizing hepatic reserve in operative candidacy. Finally, the rarity and stability of TIPS utilization reinforce that portal hypertension interventions remain tightly indication-based, even as inpatient complexity rises.

This study has several notable strengths. First, it represents one of the most extensive contemporary national analyses of hepatocellular carcinoma (HCC) in the United States, using the Nationwide Inpatient Sample (NIS). This rigorously designed, all-payer database represents over 97% of the U.S. population. The large sample size and weighted design enhance the statistical precision and generalizability of our findings. Second, by spanning five consecutive years (2018–2022), the study captures recent epidemiologic transitions, including the post-hepatitis C and early post-COVID-19 periods, providing a dynamic view of evolving etiologies. Third, strict case definitions and consistent coding algorithms were applied across years to ensure methodological uniformity and minimize misclassification bias. Fourth, the analysis separately delineated metabolic-associated steatotic liver disease (MASLD)-related and HCV-related HCC, enabling direct comparison of their temporal and clinical profiles. Finally, the use of multivariable, survey-weighted regression, adjusted for demographic and comorbidity covariates, strengthened internal validity and enabled nationally representative inference regarding inpatient outcomes.

### Study Limitations

This study also has several significant limitations. First, its retrospective and cross-sectional design precludes causal inference, and residual confounding may persist despite survey weighting and adjustment. Second, the NIS is an administrative database that lacks granular clinical data, such as tumor stage (Barcelona Clinic Liver Cancer (BCLC) classification, Child-Pugh score, or MELD score), which are key prognostic indicators in HCC and could further refine mortality risk assessment. Third, as the NIS captures hospitalizations rather than individual patients, longitudinal outcomes such as tumor recurrence, progression-free survival, and overall survival could not be assessed. Fourth, information on outpatient management, medication use, and exposure to potentially protective cardiovascular agents (e.g., statins, beta-blockers, ACE inhibitors) was not available. However, these have been associated with improved liver and cardiovascular outcomes in prior studies [23]. Finally, potential miscoding of diagnoses and procedures may lead to under- or overestimation of prevalence rates; however, such misclassification is expected to be nondifferential across study years.

## 5. Conclusions

From 2018 to 2022, inpatient resource utilization among individuals hospitalized with hepatocellular carcinoma increased significantly, primarily due to a greater burden of cardiometabolic comorbidities and the management of hepatic decompensation, rather than oncologic interventions. In-hospital mortality was mainly associated with acute severity indicators such as hepatic encephalopathy, organ failure, and sepsis, while tumor-directed procedures remained infrequent. The decreasing prevalence of viral hepatitis alongside the increasing incidence of metabolic dysfunction-associated steatotic liver disease (MASLD) indicates a fundamental epidemiologic shift with important implications for surveillance strategies and resource allocation. Current hospitalizations for hepatocellular carcinoma largely involve the management of advanced, decompensated liver disease in patients with complex medical profiles. This trend underscores the urgent need for improved outpatient surveillance focused on populations with metabolic liver disease, the implementation of early detection strategies, and the development of coordinated multidisciplinary care to prevent disease progression necessitating hospitalization.

## Figures and Tables

**Table 1:** Survey-weighted trends in mortality among patients hospitalized with hepatocellular carcinoma, 2018–2022.

Year	Mortality (%)	Standard Error	95% Confidence Interval	*p*-Value
2018	8.82%	0.002144	0.08399–0.09240	<0.001
2019	8.98%	0.002089	0.08566–0.09385	<0.001
2020	9.42%	0.002223	0.08981–0.09852	<0.001
2021	9.14%	0.002135	0.08718–0.09555	<0.001
2022	9.23%	0.002136	0.08812–0.09650	<0.001

**Table 2:** Survey-weighted temporal trends in comorbidities (percentages) among hospitalized patients with hepatocellular carcinoma, 2018–2022.

Comorbidity Burden Among HCC	2018	2019	2020	2021	2022
Cirrhosis	45.90%	45.36%	45.94%	45.15%	44.02%
Alcohol use disorder	20.18%	19.26%	19.38%	19.47%	18.15%
Viral hepatitis (HBV/HAV/HAE)	30.20%	27.89%	25.43%	23.32%	20.73%
HCV	26.47%	24.34%	21.79%	19.58%	17.09%
COVID-19	-	-	2.3%	3.1%	5.2%
MASLD	6.640%	7.370%	7.610%	8.150%	8.740%
Diabetes mellitus	35.95%	36.74%	36.29%	37.72%	38.89%
Hypertension	60.31%	62.15%	62.35%	63.55%	63.90%
Obesity	10.56%	11.30%	12.25%	12.48%	13.69%
Chronic kidney disease (CKD)	18.05%	19.46%	19.96%	20.06%	20.01%
Congestive heart failure (CHF)	12.01%	13.25%	14.07%	14.53%	15.49%
Coronary artery disease (CAD)	18.40%	19.50%	20.03%	19.96%	19.91%
Dyslipidemia	26.42%	28.68%	30.30%	32.81%	34.36%
Morbid obesity	3.440%	3.660%	3.760%	3.890%	4.480%

Values represent survey-weighted percentages. Statistical testing was not performed, as the table is intended to describe population-level temporal patterns. Abbreviations: MASLD, metabolic dysfunction-associated steatotic liver disease; HCC, hepatocellular carcinoma; HCV, Hepatitis C Virus.

**Table 3:** Survey-weighted prevalence (percentages) of major admission drivers among hospitalizations involving hepatocellular carcinoma, 2018–2022.

Year	Liver-Related (%)	Infectious (%)	Cardiovascular (%)	Renal (%)	GI Bleeding (%)
2018	56.10%	40.10%	40.00%	32.00%	17.20%
2019	55.50%	41.30%	41.80%	32.70%	16.80%
2020	56.10%	42.50%	44.50%	33.20%	18.10%
2021	55.40%	43.40%	44.40%	33.00%	18.30%
2022	54.60%	42.90%	45.80%	32.60%	17.90%

Values represent survey-weighted percentages (%). Admission categories are not mutually exclusive. Abbreviations: GI Bleeding, gastrointestinal bleeding.

**Table 4:** Adjusted predictors of mortality among patients hospitalized with hepatocellular carcinoma in the United States, 2018–2022.

Predictors of In-Hospital Mortality, Adjusted Odds Ratios (aOR)	2018 (aOR)	2019 (aOR)	2020 (aOR)	2021 (aOR)	2022 (aOR)
**Demographics**					
Age (per year)	1.018	1.021	1.025	1.017	1.022
Female Sex	0.838	0.978	0.749	0.957	0.823
**Race/Ethnicity**					*p* < 0.001
African American	1.300	1.462	1.393	1.117	1.473
Hispanic	0.941	0.870	0.880	1.040	0.849
Asian/Pacific Islander	1.232	1.065	0.814	1.295	0.993
Native American	1.062	1.803	1.178	1.247	1.310
Other Race	1.256	1.332	1.327	1.162	0.968
**Insurance/Socioeconomics**					*p* < 0.001
Medicaid	1.202	1.589	1.39	1.204	1.096
Private Insurance	1.377	1.497	1.306	1.267	1.228
Self-pay	1.637	2.242	2.134	1.488	1.397
Other Payer	1.033	2.205	0.733	0.378	1.758
**Clinical Predictors of Mortality**					
**Liver Disease**					*p* < 0.001
Liver Failure	2.747	2.677	2.513	2.641	2.519
Hepatic Encephalopathy	6.852	8.542	11.126	7.397	8.871
SBP	1.364	1.05	1.118	1.187	0.963
Ascites	1.214	1.12	1.216	1.170	1.137
Hepatorenal Syndrome	2.668	3.366	2.173	2.284	2.784
**Renal and Infection-Related Events**					*p* < 0.001
Acute Kidney Injury	1.448	1.373	1.480	1.311	1.309
Sepsis	3.584	3.861	4.215	4.278	3.873
COVID-19(Primary Diagnosis)	-	-	2.688	2.610	0.960
**Gastrointestinal Bleeding Events**					*p* < 0.001
Non-variceal UGI Bleed	1.755	2.120	1.743	1.831	2.103
Variceal UGI Bleed	1.619	1.501	1.315	1.603	1.572
Lower GI Bleed	1.161	1.046	1.150	1.126	0.993
**Cardiovascular and Thrombotic Events**					*p* < 0.001
Congestive Heart Failure	1.423	1.277	1.2	1.325	1.191
Coronary Artery Disease	0.995	1.155	1.207	1.034	1.149
Stroke	2.108	1.482	1.389	1.330	1.593
Atrial Fibrillation	1.391	1.259	1.216	1.116	1.234
Pulmonary Embolism	2.367	2.206	2.424	1.666	1.675
Acute DVT	1.267	1.038	0.937	1.109	1.313
Portal Vein Thrombosis	1.451	1.12	0.963	1.006	1.215
**Metabolic**					*p* < 0.001
MASLD	0.700	0.734	0.666	0.689	0.800
Obese	1.830	1.565	1.061	0.691	0.810
Morbidly Obese	1.353	1.298	1.398	1.515	1.234
**Hospital Characteristics**					*p* < 0.001
Bed Size—Medium	0.959	0.937	0.840	0.953	1.087
Bed Size—Large	0.821	0.751	0.816	0.800	0.957
Urban non-teaching	0.527	0.532	0.889	0.728	0.724
Urban Teaching	0.471	0.513	0.732	0.566	0.694
Midwest	0.917	0.926	0.698	0.831	0.716
South	0.800	0.790	0.741	0.666	0.731
West	0.981	0.923	0.805	0.668	0.809

Values represent adjusted odds ratios (aORs) from survey-weighted multivariable logistic regression.

**Table 5:** Survey-weighted length of stay and total hospital charges across primary admission categories among HCC hospitalizations, 2018–2022.

Length of Stay (Days)	2018	2019	2020	2021	2022
Liver-related	6.4	6.37	6.72	6.78	7.22
Infectious	8.6	8.36	9.49	10.25	9.55
Cardiovascular	5.07	5.12	5.51	5.8	5.79
Renal	5.65	5.64	5.88	6.08	6.14
GI bleed/Ulcer	7.32	7.46	7.86	8.28	8.32
Other	3.81	3.83	3.91	4.04	4.03
**Total Hospital Charges** **(USD)**					
Liver-related	$72,333	$74,943	$84,814	$87,957	$96,701
Infectious	$111,892	$115,280	$137,974	$156,176	$142,495
Cardiovascular	$65,454	$69,792	$77,996	$84,484	$87,152
Renal	$63,007	$66,148	$72,155	$76,742	$79,719
GI bleed/Ulcer	$112,562	$120,991	$132,947	$143,316	$147,014
Other	$37,334	$39,721	$41,914	$44,849	$46,815

**Table 6:** Survey-weighted temporal trends in demographics and socioeconomic characteristics among hospitalized patients with HCC, 2018–2022.

Characteristics	2018	2019	2020	2021	2022
**Demographics**					
Mean age (years)	64.4	65.0	65.2	65.6	65.8
**Sex**					
Male	67.10%	67.10%	66.80%	65.40%	65.90%
Female	32.90%	32.90%	33.20%	34.60%	34.10%
**Race**					
White	59.40%	60.00%	60.80%	59.90%	59.90%
African American	13.50%	13.60%	13.10%	13.30%	12.40%
Hispanic	16.10%	15.80%	15.80%	16.30%	17.10%
Asian/Pacific Islander	6.30%	6.50%	5.90%	6.50%	6.40%
Native American	0.90%	0.80%	0.80%	0.90%	0.90%
Other	3.80%	3.30%	3.50%	3.20%	3.30%
**Payer**					
Medicare	54.00%	55.60%	55.90%	56.40%	58.00%
Medicaid	17.00%	15.80%	15.90%	15.50%	14.00%
Private	22.80%	22.10%	21.80%	21.70%	21.90%
Self-pay	2.70%	2.80%	2.60%	2.70%	2.30%
Other	0.20%	0.20%	0.30%	0.20%	0.20%
**Region**					
Northeast	19.20%	19.40%	19.60%	19.80%	19.10%
Midwest	19.70%	19.60%	19.50%	19.30%	19.30%
South	37.70%	37.80%	38.30%	38.50%	38.50%
West	23.40%	23.20%	22.60%	22.40%	23.20%
**Hospital Characteristics**					
Rural	4.20%	4.40%	4.70%	4.70%	4.80%
Urban, non-teaching	14.90%	13.30%	13.20%	13.40%	12.50%
Urban, teaching	80.90%	82.30%	82.10%	82.00%	82.70%
**Bed Size**					
Small	15.30%	16.20%	17.10%	17.80%	16.90%
Medium	26.30%	25.20%	25.30%	24.70%	25.90%
Large	58.40%	58.60%	57.60%	57.50%	57.20%
**Income Quartile**					
1 (lowest)	29.90%	30.10%	29.30%	29.90%	28.60%
2	25.90%	24.40%	26.50%	24.60%	24.30%
3	23.10%	24.20%	23.10%	23.90%	24.70%
4 (highest)	21.20%	21.30%	21.00%	21.60%	22.40%
**Admission Day**					
Weekday	78.40%	78.70%	79.10%	79.50%	78.20%
Weekend	21.60%	21.30%	20.90%	20.50%	21.80%

Values are presented as survey-weighted means or percentages (%) as appropriate. Statistical testing was not performed; values describe population-level trends.

**Table 7:** Survey-weighted percentage of hospitalized patients with hepatocellular carcinoma who underwent endoscopic and hepatobiliary procedures during hospitalization, 2018–2022.

Procedure Type	2018	2019	2020	2021	2022	*p*-Value
**Endoscopic procedures**						
Diagnostic EGD	7.3%	7.23%	7.33%	7.8%	8.14%	<0.01
Interventional EGD	3.7%	3.9%	4.06%	3.94%	3.97%	0.41
Total EGD	10.2%	10.37%	10.59%	10.92%	11.23%	<0.01
Total Colonoscopy	2.58%	2.52%	2.54%	2.63%	2.91%	0.08
Total ERCP	5.59%	6.04%	6.34%	6.36%	6.47%	<0.01
**Hepatobiliary/oncologic procedures**						
TIPS	0.25%	0.26%	0.24%	0.17%	0.20%	0.48
Liver transplantation	3.06%	2.71%	2.82%	2.54%	2.66%	0.09
Hepatic resection	8.58%	8.14%	8.34%	8.55%	8.32%	0.77

Values represent the survey-weighted percentage (%) of hospitalizations in which each procedure was performed.

**Table 8:** Adjusted odds ratio predictors of inpatient liver transplantation, hepatic resection, and TIPS among hospitalized patients with hepatocellular carcinoma, 2018–2022.

Predictor of Inpatient Liver Transplantation	2018 (a OR)	2019 (a OR)	2020 (a OR)	2021 (a OR)	2022 (a OR)	*p* Value
Age (per year)	0.97	0.97	0.97	0.97	0.97	<0.001
Female sex	0.92	0.95	0.88	0.93	0.90	0.18
**Race/Ethnicity**						
African American	0.42	0.38	0.41	0.60	0.58	<0.001
Hispanic	1.12	1.18	1.22	1.30	1.26	0.11
Asian / Pacific Islander	1.05	1.10	1.08	0.98	1.02	0.74
Another race	0.80	0.76	0.83	0.90	0.88	0.21
**Insurance type**						
Medicaid	0.55	0.48	0.52	0.56	0.71	<0.001
Private insurance	1.52	1.48	1.45	1.48	1.60	<0.001
Self-pay	0.28	0.22	0.25	0.34	0.07	<0.001
**Comorbidity**						
MASLD	3.87	3.20	3.45	2.26	2.86	<0.001
Chronic hepatitis C	2.33	2.01	1.92	1.45	1.35	<0.001
Alcohol-associated liver disease	1.84	1.73	1.68	1.52	1.41	<0.001
Cirrhosis	3.10	3.40	3.25	2.82	3.18	<0.001
Substance use disorder	0.28	0.31	0.29	0.25	0.4	<0.001
**Hospital Type**						
Urban teaching hospital	20.4	9.8	7.3	6.9	2.5	<0.001
**Predictor of Inpatient Hepatic Resection**						
Age (per year)	0.98	0.98	0.99	0.99	0.98	<0.001
MASLD	1.35	1.28	1.30	1.22	1.40	<0.001
Obesity	1.48	1.44	1.31	1.50	1.35	<0.001
Dyslipidemia	1.22	1.20	1.18	1.19	1.18	0.006
Cirrhosis	0.60	0.58	0.62	0.62	0.55	<0.001
Chronic kidney disease	0.72	0.75	0.78	0.74	0.65	<0.001
Urban teaching hospital	2.35	2.28	2.15	2.21	2.29	<0.001
**Predictor of TIPS**						
Cirrhosis	18.7	16.2	14.8	16.1	16.4	<0.001

Values represent adjusted odds ratios (aORs) for inpatient liver transplantation, hepatic resection, and TIPS placement.

## Data Availability

The data analyzed in this study are publicly available from the Healthcare Cost and Utilization Project National Inpatient Sample (NIS).

## Abbreviations

The following abbreviations are used in this manuscript:

a OR	Adjusted odds ratio
AKI	Acute kidney injury
AUD	Alcohol use disorder
CHF	Congestive heart failure
CI	Confidence interval
COVID-19	Coronavirus disease 2019
DAA	Direct-acting antiviral
EGD	Esophagogastroduodenoscopy
ERCP	Endoscopic retrograde cholangiopancreatography
GI	Gastrointestinal
HCC	Hepatocellular carcinoma
HCUP	Healthcare Cost and Utilization Project
HCV	Hepatitis C virus
HRS	Hepatorenal syndrome
ICD-10-CM	International Classification of Diseases, Tenth Revision, Clinical Modification
LOS	Length of stay
MASLD	Metabolic dysfunction–associated steatotic liver disease
NIS	National Inpatient Sample
OR	Odds ratio
SE	Standard error
TIPS	Transjugular intrahepatic portosystemic shunt
USD	United States dollars
